# "The West London Appointment": an Official Disclaimer

**Published:** 1910-12-31

**Authors:** Donald Armour

**Affiliations:** Hon. Sec. to the Medical Staff.


### Editor's Letter-Box.

"THE WEST LONDON APPOINTMENT."
AN OFFICIAL DISCLAIMER.

*To the Editor of* The Hospital.

West London Hospital,
Hammersmith Road, W.,
December 19, 1910.

Dear Sir,—I am desired by the medical staff of the West London Hospital to forward you the enclosed letter, which they trust you will insert in the next issue of The Hospital.—Yours faithfully, Donald Armour,
Hon. Sec. to the Medical Staff.

